# Thymic carcinoma metastasize to the small intestine: a case report

**DOI:** 10.1186/s12876-020-01505-7

**Published:** 2020-10-28

**Authors:** Yi Yuan, Hong Pu, Ming-hui Pang, Yi-sha Liu, Hang Li

**Affiliations:** 1 Department of Radiology, Sichuan Academy of Medical Sciences and Sichuan Provincial People’s Hospital, 32# Second Section of First Ring Road, Qingyang District, Chengdu, 610070 Sichuan China; 2 Department of Gastrointestinal Surgery, Sichuan Academy of Medical Sciences and Sichuan Provincial People’s Hospital, 32# Second Section of First Ring Road, Qingyang District, Chengdu, 610070 Sichuan China; 3 Department of Pathology, Sichuan Academy of Medical Sciences and Sichuan Provincial People’s Hospital, 32# Second Section of First Ring Road, Qingyang District, Chengdu, 610070 Sichuan China

**Keywords:** Thymoma, Intestinal neoplasms, Diagnosis, Differential, Carcinoma, Squamous cell, Biopsy

## Abstract

**Background:**

Thymic carcinoma is a rare mediastinal neoplasm with a high malignant potential. It often shows pleural invasion and distant metastasis. The metastasis of thymic carcinoma to the small intestine is rarely reported and difficult to distinguish from other gastrointestinal tract tumors.

**Case presentation:**

An elderly man presented with lower abdominal pain for 2 months. Abdominal CT showed a mass communicated with the small intestinal lumen. After radical resection of the small intestinal tumor, resected specimens showed moderately differentiated squamous-cell carcinoma with lymph nodes metastases. The patient received chest CT and was found to have a mass in anterior mediastinum. Biopsies of the mass revealed thymic squamous-cell carcinoma.

**Conclusions:**

We highlighted the metastasis of thymic carcinoma to the small intestine is rare and easily misdiagnosed. In patients with a mass communicated with the small intestinal lumen, a suspicion of thymic carcinoma metastasis should not be overlooked and we should make accurate differential diagnosis from the other small intestinal tumors.

## Background

In the anterosuperior mediastinum, thymic neoplasms mainly include thymoma and thymic carcinoma. Thymic carcinoma is a rare mediastinal neoplasm and has been reported to comprise only 0.06% of all thymic neoplasms [1]. Thymic carcinoma has a high malignant potential with a tendency to distant metastasis [2]. 80% of cases have local invasion of contiguous mediastinal structures, and 40% of cases present metastatic spread to bones, lung, pleura, liver, or lymph node [3]. However, the metastasis of thymic carcinoma to the small intestine is extremely rare. It has the similar imaging characteristics with the other tumors, such as gastrointestinal stromal tumor (GIST) and could be easily misdiagnosed. In this paper, we presented a case of an elderly man with an initial diagnosis of GIST, but histopathology indicated the metastasis of thymic carcinoma to the small intestine.

## Case presentation

**Fig. 1:**
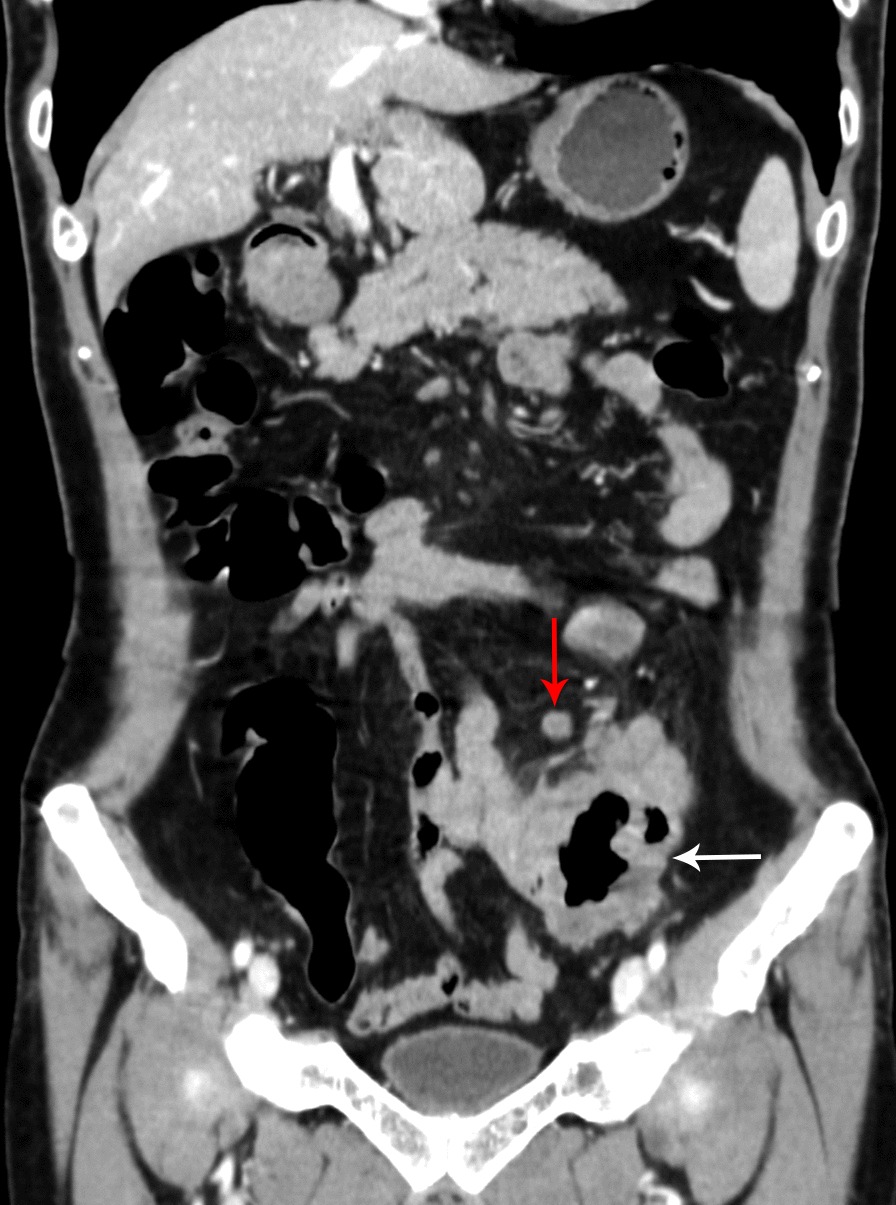
Abdominal CT. Abdominal CT shows a mass communicated with the small intestinal lumen (white arrow), and the outer and inner margins of the mass are irregular. A lymph node involvement is observed in the adjacent mesentery (red arrow)

**Fig. 2:**
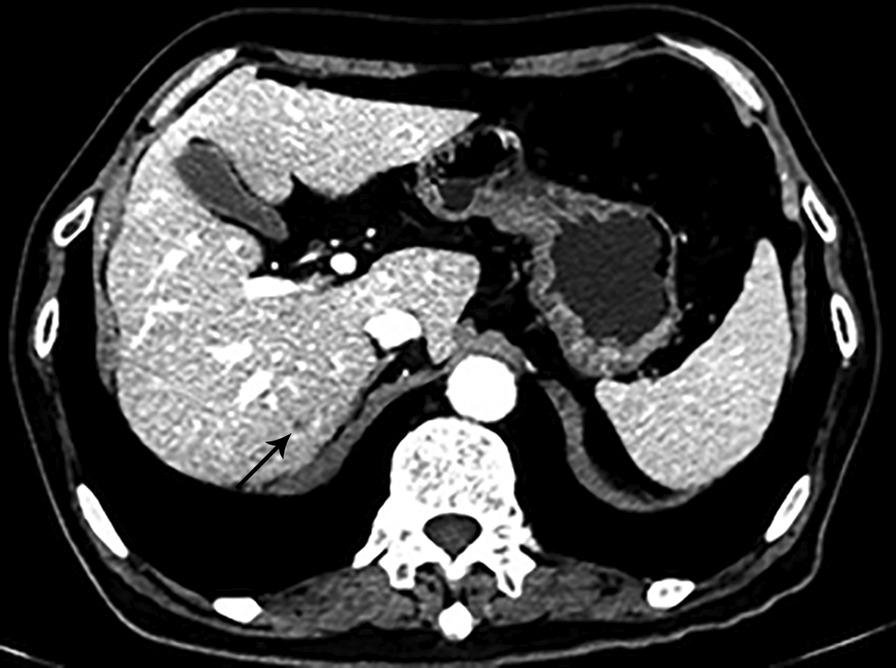
Abdominal CT. After enhancement, abdominal CT shows a small low-density lesion in the seventh segment of the liver (black arrow)

**Fig. 3:**
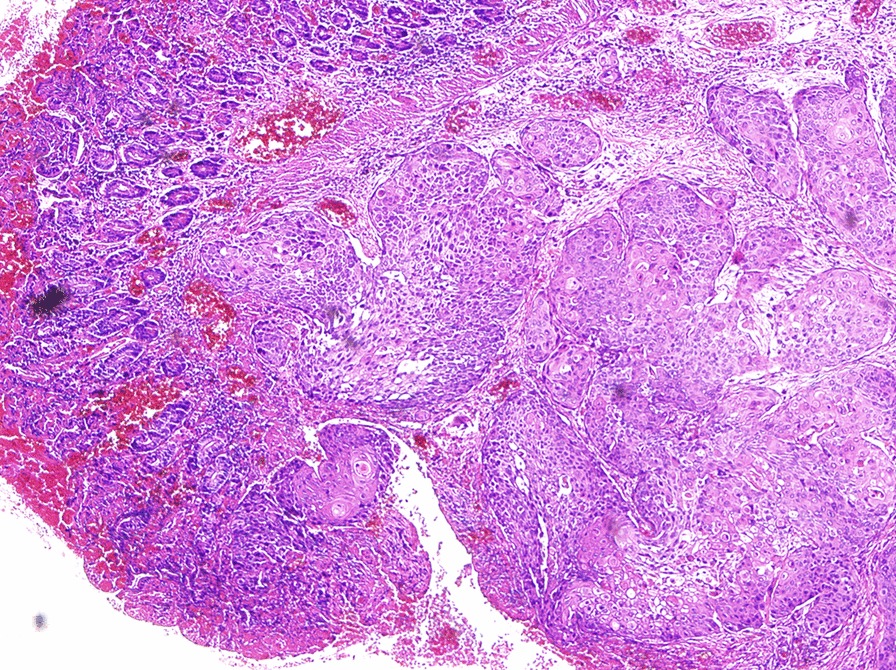
Pathologic findings. Microscopic examination of the surgical specimens reveals squamous cell carcinoma infiltrating the entire intestinal wall (original magnification × 100; HE stain)

**Fig. 4:**
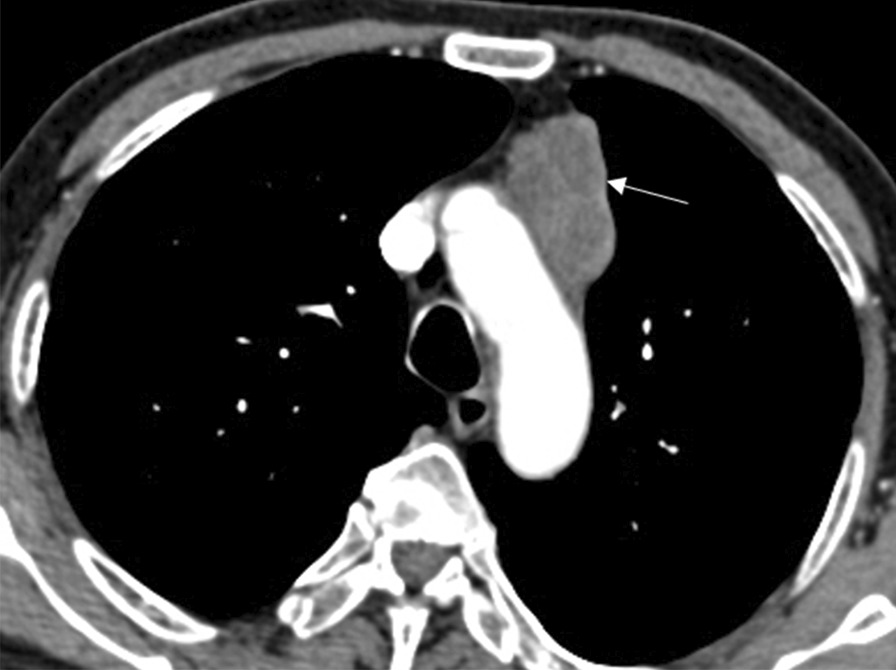
Chest CT. Chest CT shows an irregular contours mass in anterior mediastinum with mild heterogenetic enhancement (white arrow)

A 72-year-old man presented with lower abdominal pain for 2 months, accompanied by bloody stools occasionally. On physical examination, there was a soft abdomen with mild tenderness in the left lower quadrant. In palpation, a mass was identified in the lower abdomen, measuring 4 × 3 cm approximately. The mass boundary was unclear and the mass can be mobile. The patient had no family history. Laboratory examination revealed hemoglobin (HGB) of 85 g/L, leucocytes of 15.41 × 10^9^/L and K of 3.28 mmol/L. Colonoscopy showed no abnormalities. Abdominal CT shows a 4.7 × 3.7 cm mass communicated with the small intestinal lumen (Fig. 1) and the edge of the mass was rough, with shallow lobes. Several enlarged lymph nodes were observed in the adjacent mesentery and in the right cardiophrenic angle. After enhancement, the mass shows heterogenetic enhancement and a small lesion in the seventh segment of the liver (Fig. 2). Based on CT characteristic, GIST was first considered. Subsequently, the patient underwent radical resection of the small intestinal tumor and intraoperative liver lesion ablation. At laparotomy, the mass was covered in omentum majus and invaded the adjacent peritoneum. Multiple enlarged lymph nodes were observed in mesentery with the largest diameter of 2 cm and three small lesions were observed in liver with the largest diameter of 0.5 cm. Histopathology of the small intestinal tumor and liver lesion showed moderately differentiated squamous-cell carcinoma with lymph nodes metastases. Microscopic examination of the surgical specimens indicates squamous cell carcinoma infiltrating the entire intestinal wall (Fig. 3). Immunohistochemical investigations demonstrated positive for cytokeratin 19, cytokeratin 5/6, P63and Ki-67 (approximately 30%). However, immunohistochemistry indicates negative for CD5 and CD117. The patient subsequently received chest CT and was found to have an irregular contours mass in anterior mediastinum with mild heterogenetic enhancement, measuring 2.3 × 1.4 cm (Fig. 4). Biopsies of the anterior mediastinum mass revealed thymic squamous-cell carcinoma. Although both CD5 and CD117 for intestinal mass were negative, after multidisciplinary consultation, clinician concluded the small intestine mass was metastasized from the thymus because primary small intestinal squamous cell carcinoma is extremely rare, and both thymic and small intestinal squamous cell carcinoma were confirmed by pathological examination. The patient was started on gemcitabine plus cisplatin regimen.

## Discussion and conclusions

Most thymic carcinomas present initially with cough, fatigue, chest pain, fever, loss of appetite, and weight loss [4]. Thymic carcinoma is a rare carcinoma of the thymus arising in the thymic epithelium. It has the similar malignant characteristics with other organ, which tend to have capsular invasion and metastases [5].Previous study reported one-third of thymic carcinoma patients had lymph node involvement or distant metastasis [6]. The incidence of extrathoracic metastases for thymic carcinoma is approximately 3–6%, which makes diagnosis difficult [7]. Most of the thymic carcinomas often metastasize to bones, lung, pleura, liver, or lymph nodes [3, 5]. There were no specific signs for the metastasis of thymic carcinoma. Previous studies reported lymph node metastasis was common and the anterior lymph nodes were first involved, with subsequent progression to the intrathoracic lymph nodes, and then the extrathoracic lymph nodes [6]. Bone metastasis were characterized by osteolytic bone destruction combined with soft tissue mass formation [8]. Pleural and lung metastasis was present as multiple small nodules. MRI of the brain with intravenous gadolinium showed heterogenetic enhancing nodule with central necrosis and large edema surrounding [9]. To our knowledge, the metastasis of thymic carcinoma to the small intestine was extremely rare and only one study had reported in the literature [7].

There are limited data about the incidence of the small intestinal metastasis in patients with thymic carcinoma and the mechanism of distant metastasis from thymic carcinoma to small intestine remains unclear. Previous study suggested that extrathoracic metastases may occur because the tumor cells penetrated and spread from the great vessels in thymic carcinoma [2]. In our case, we indicated that hematogenous metastasis is the primary pathway leading to the metastasis of thymic carcinoma to the small intestine. Differential diagnoses for small intestinal masses included metastatic lesion, GIST, lymphoma and adenocarcinoma. GIST is the most common mesenchymal tumor in the gastrointestinal tract [10]. GIST typically presents as a submucosal tumor of the gastrointestinal wall, occasionally accompanied by mucosal ulcer and rupture of tumor [11]. Patients may have hematemesis, melena, hematochezia, or signs and symptoms of anemia [12]. Cavity and fistula formation may occur, which results in luminal enlargement and communication of the cavity or fistula with the small intestinal lumen [12]. The outer margins of GIST are typically sharply defined and the inner margins may show smooth. Moreover, GIST seldom had regional lymph nodes metastasis [13]. Our case showed the irregular outer and inner margins of the mass, and lymph node involvement in the adjacent mesentery and right cardiophrenic angle. Lymphoma is the second most common tumor of the small intestine neoplasm [14]. Lymphoma usually presents as a homogeneous soft tissue mass without necrosis. The intestinal lymphoma often has thickened wall and presents as aneurysmal dilatation.[15]. Lymphoma often shows mild enhancement and preservation of the fat plane [16]. And the inner margins of lymphoma are smooth, unlike as the mass presenting with irregular inner margins in our case. In addition, lymphoma rarely present with liver metastasis. Intestinal adenocarcinoma typically shows luminal narrowing, which may result in intestinal obstruction.

As regard to the treatment for thymic carcinoma, surgery remains the mainstay of treatment, and radiation and chemotherapy also have been applied widely as adjuvant and palliative procedures [17]. For resectable patients, total thymectomy and complete tumor excision is recommended [18]. For patients with metastatic thymic carcinoma, platinum-based chemotherapy is recommended for first-line therapy [18]. Therefore, the patient was started on gemcitabine plus cisplatin regimen.

In conclusion, the metastasis of thymic carcinoma to the small intestine is rare and easily misdiagnosed. When a mass communicated with the small intestinal lumen is seen on CT, a suspicion of metastatic small intestinal neoplasms should not be overlooked and we should make accurate differential diagnosis from the other small intestinal tumors. Abdominal and chest CT is helpful to make the preoperative diagnosis and accurate treatment.

## Supplementary information

**Additional file 1**. CARE Checklist of Information.

## Data Availability

Not applicable.

## Abbreviations

CT: Computed tomography
GIST: Gastrointestinal stromal tumor
HGB: Hemoglobin
MRI: Magnetic resonance imaging
