# Supplementary material for: Thymic carcinoma metastasize to the small intestine: a case report
Source: BMC Gastroenterol. 2020 Oct 28;20:358. doi: 10.1186/s12876-020-01505-7 (PMC7594467; doi:10.1186/s12876-020-01505-7)
Supplement: Supplementary file 1 — Additional file 1. CARE Checklist of Information. [file 12876_2020_1505_MOESM1_ESM.pdf]

CARE Checklist of information to include when writing a case report

CARE case report guidelines

Topic	Item	Checklist item description	Reported on Line
**Title**	1	The diagnosis or intervention of primary focus followed by the words "case report"	Thymic Carcinoma Metastasize to The Small Intestine: case report
**Key Words**	2	2 to 5 key words that identify diagnoses or interventions in this case report, including "case report"	Thymoma; Intestinal Neoplasms; Diagnosis, Differential; Carcinoma, Squamous Cell; Biopsy
**Abstract (no references)**	3a	Introduction: What is unique about this case and what does it add to the scientific literature?	Introduction, paragraph 1
	3b	Main symptoms and/or important clinical findings	Case presentation, paragraphy 1
	3c	The main diagnoses, therapeutic interventions, and outcomes	Case presentation, paragraphy 1
	3d	Conclusion—What is the main "take-away" lesson(s) from this case?	Discussion and conclusion, paragraph 5
**Introduction**	4	One or two paragraphs summarizing why this case is unique (**may include references**)	Introduction, paragraph 1; Discussion and conclusion, paragraph 1
**Patient Information**	5a	De-identified patient specific information.	Not applicable
	5b	Primary concerns and symptoms of the patient.	Case presentation, paragraphy 1
	5c	Medical, family, and psycho-social history including relevant genetic information	Case presentation, paragraphy 1
	5d	Relevant past interventions with outcomes	Case presentation, paragraphy 1
**Clinical Findings**	6	Describe significant physical examination (PE) and important clinical findings.	Case presentation, paragraphy 1
**Timeline**	7	Historical and current information from this episode of care organized as a timeline	Not applicable
**Diagnostic Assessment**	8a	Diagnostic testing (such as PE, laboratory testing, imaging, surveys).	Case presentation, paragraphy 1
	8b	Diagnostic challenges (such as access to testing, financial, or cultural)	Case presentation, paragraphy 1
	8c	Diagnosis (including other diagnoses considered)	Case presentation, paragraphy 1
	8d	Prognosis (such as staging in oncology) where applicable	Not applicable
**Therapeutic Intervention**	9a	Types of therapeutic intervention (such as pharmacologic, surgical, preventive, self-care)	Case presentation, paragraphy 1
	9b	Administration of therapeutic intervention (such as dosage, strength, duration)	Not applicable
	9c	Changes in therapeutic intervention (with rationale)	Case presentation, paragraphy 1
**Follow-up and Outcomes**	10a	Clinician and patient-assessed outcomes (if available)	Not applicable
	10b	Important follow-up diagnostic and other test results	Case presentation, paragraphy 1
	10c	Intervention adherence and tolerability (How was this assessed?)	Not applicable
	10d	Adverse and unanticipated events	Not applicable
**Discussion**	11a	A scientific discussion of the strengths AND limitations associated with this case report	Not applicable
	11b	Discussion of the relevant medical literature **with references**.	Discussion and conclusion, paragraph 2-4
	11c	The scientific rationale for any conclusions (including assessment of possible causes)	Discussion and conclusion, paragraph 2

11d The primary “take-away” lessons of this case report (without references) in a one paragraph conclusion . . . . Discussion and conclusion, paragraph 5

.

Patient Perspective **12** The patient should share their perspective in one to two paragraphs on the treatment(s) they received . . . . Not applicable

Informed Consent **13** Did the patient give informed consent? Please provide if requested . . . . **Yes**√☐ **No** ☐
